# A novel method to detect unlabeled inorganic nanoparticles and submicron particles in tissue by sedimentation field-flow fractionation

**DOI:** 10.1186/1743-8977-5-18

**Published:** 2008-12-03

**Authors:** Cassandra E Deering, Soheyl Tadjiki, Shoeleh Assemi, Jan D Miller, Garold S Yost, John M Veranth

**Affiliations:** 1 Department of Pharmacology & Toxicology, University of Utah, 30 South 2000 East, Salt Lake City, Utah, USA; 2 Postnova Analytics USA, 230 South 500 East, Suite 150, Salt Lake City, Utah 84103, USA; 3 Department of Metallurgical Engineering, University of Utah, 135 South 1460 East, Salt Lake City, 84112 Utah, USA

## Abstract

A novel methodology to detect unlabeled inorganic nanoparticles was experimentally demonstrated using a mixture of nano-sized (70 nm) and submicron (250 nm) silicon dioxide particles added to mammalian tissue. The size and concentration of environmentally relevant inorganic particles in a tissue sample can be determined by a procedure consisting of matrix digestion, particle recovery by centrifugation, size separation by sedimentation field-flow fractionation (SdFFF), and detection by light scattering.

**Background:**

Laboratory nanoparticles that have been labeled by fluorescence, radioactivity, or rare elements have provided important information regarding nanoparticle uptake and translocation, but most nanomaterials that are commercially produced for industrial and consumer applications do not contain a specific label.

**Methods:**

Both nitric acid digestion and enzyme digestion were tested with liver and lung tissue as well as with cultured cells. Tissue processing with a mixture of protease enzymes is preferred because it is applicable to a wide range of particle compositions. Samples were visualized via fluorescence microscopy and transmission electron microscopy to validate the SdFFF results. We describe in detail the tissue preparation procedures and discuss method sensitivity compared to reported levels of nanoparticles *in vivo*.

**Conclusion:**

Tissue digestion and SdFFF complement existing techniques by precisely identifying unlabeled metal oxide nanoparticles and unambiguously distinguishing nanoparticles (diameter<100 nm) from both soluble compounds and from larger particles of the same nominal elemental composition. This is an exciting capability that can facilitate epidemiological and toxicological research on natural and manufactured nanomaterials.

## Background

The toxicology of nano-sized (diameter < 100 nm) particles is a topic of current interest because there have been rapid advances in the synthesis of novel nanomaterials for research, consumer, and industrial applications. Recent reviews have discussed nanoparticle health effects [1,2]. The growing evidence of adverse health effects from exposure to incidentally produced ultrafine particles from combustion and atmospheric processes motivates concern about manufactured nanomaterials. There is epidemiological evidence for cardiovascular effects of ambient ultrafine particulate matter (PM) [3]. Indications that inhaled particles can translocate to the other organs [4] suggest a link between nanoparticles and neurodegenerative diseases [5] and other systemic pathologies. Monitoring human exposure to engineered nanoparticles (from air, water, food, consumer products, and soil), determining the rate of particle uptake by humans and food chain organisms, and measuring the resulting nanoparticle concentrations in target organs are major challenges for nanoparticle toxicology studies [6].

Most nanoparticle uptake and translocation research has quantified nanoparticles *in vivo *using some type of unique particle label. For example, nanoparticle laboratory studies have included radioactive particles [4], trace metals such as gold and iridium [7], and fluorescent particles [8]. However, the population exposures most relevant to health involve the emissions or deliberate release of high-production-volume manufactured nanomaterials and exposures to incidental nanoparticles, such as soot. Combustion emissions and manufactured powders such as fumed silica, ultrafine titanium dioxide (TiO_2_), and similar industrial materials rarely have a unique and easily detected label.

Examples of current techniques for measuring unlabeled inorganic nanoparticles in animal organs include using electron microscopy to show localization of TiO_2 _particles to the lung of rats [9] and using elemental analysis to show the presence of manganese particles in neural tissue [10]. However, measuring changes in the concentration of unlabeled particles in tissue with these techniques is difficult. Extracting quantative information from TEM images is inexact and elemental analysis does not distinguish particles from soluble forms and provides no information on particle size.

A promising method to measure size and concentration of unlabeled nanoparticles is through separation by field-flow fractionation (FFF), which was first developed in the 1960s for separating macromolecules, colloids, and particles [11,12]. FFF has been used for the measurement of numerous properties of macromolecules and colloidal particles, including particle mass, size, and density. Caldwell *et al*. reported seminal work applying FFF to detect protein-based particles in eye lens cataracts [13]. FFF has been used to characterize natural aquatic colloids [14-16], and perform size separation of single-walled carbon nanotubes [17].

FFF is similar to chromatography methods in that materials are separated by transport velocity, but in place of a retention media the separation is carried out in a thin, open channel with bulk flow in the longitudinal direction and a separation field (centrifugal force, electric field, thermal gradients, or cross-flow) in the perpendicular direction. Particles are driven to the wall by the separation field and average particle distance from the wall is determined by the competition between the separation field and the size-dependent diffusion of particles against the concentration gradient. Since the narrow channel has a parabolic flow profile (laminar flow), the particles farthest from the wall are in the highest velocity streamlines and therefore travel the fastest. Sedimentation FFF (SdFFF) uses centrifugal force to generate the separation field. The minimum detectable particle size depends on the particle density and the maximum centrifugal force of the SdFFF instrument [18,19]. Giddings provides a full derivation of the governing equation and a graph of minimum resolvable diameter versus GΔρ for a typical instrument channel geometry [20]. For example, with the instrument used in this study, silica particles with a density of 2.0–2.65 g/ml and as small as ~22 nm can be separated using the instrument's maximum centrifugal force. For denser particles such as gold, applying the same field can separate particles as small as 10 nm. Multiple detection techniques can be used simultaneously with FFF, including fluorescence, ultra-violet absorption, and light scattering.

In this study tissue lysis and gradient centrifugation, well-established methodologies for the density-dependent separation of subcellular fractions, were adapted to isolate oxide particles from biological samples. Particle isolation was combined with SdFFF to detect and quantify unlabeled inorganic nanoparticles.

## Results

Preliminary experiments were conducted to calibrate the instrumentation used in this study and to determine the amount of particles needed for reliable detection. We used dilutions prepared from purchased silicon dioxide (SiO_2_) standards (Postnova Analytics) with a known particle size, 70 nm, and a starting concentration of 25 mg/ml of particles suspended in aqueous surfactant. With the available light scattering detector, reliable quantification of the standard could be obtained with as few as 7 × 10^10 ^particles per injected sample, which is equivalent to 25 μg of particle mass. Based on these data, our subsequent experimental work used tissue samples containing 1–2 mg of particles. Using particle aliquots greater than 40 times the limit of detection enabled robust quantification in the experiments to develop nanoparticle recovery protocols.

**Figure 1:**
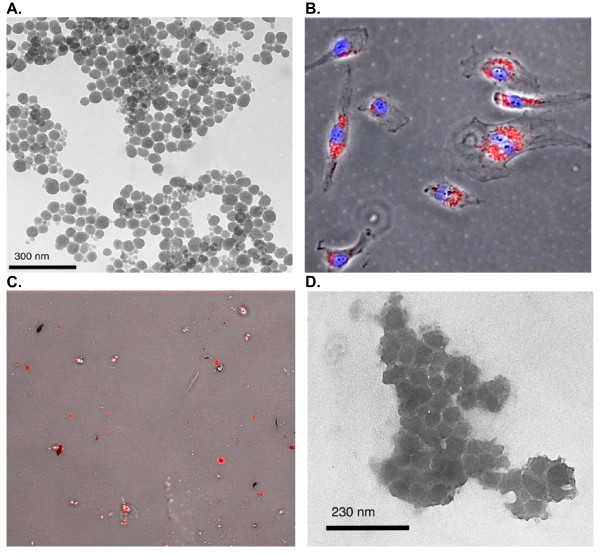
A. TEM image of the as-received 70-nm rhodamine labeled SiO_2 _particles. B. Fluorescence microscope image of human aortic endothelial cells (HAECs) treated with 70-nm rhodamine labeled SiO_2 _particles (10 μg/cm^2^) for 24 hrs. 20× objective, 200× magnification. Red= SiO_2 _particles; Blue = DAPI stained nucleus. C. Fluorescence image of cell lysate containing the 70-nm rhodamine labled particles. 20× objective, 200× magnification. D. TEM image of a dried aliquot of the final sample after cleanup for SdFFF containing 70-nm rhodamine labeled SiO_2 _particles in dried residual material.

To allow comparison of the SdFFF results to established techniques, we performed fluorescence microscopy and TEM on particle-treated cell culture samples treated with rhodamine-labeled SiO_2 _particles and prepared by enzyme digestion for SdFFF analysis. Figure 1A shows the starting 70-nm rhodamine labeled particles in aqueous surfactant visualized using TEM. The TEM confirms that the manufacturer's size is correct. Figure 1B demonstrates the interaction of 70-nm rhodamine labeled particles with Human Aortic Endothelial Cells (HAECs). This image shows localization of the nanoparticles to the cells and formation of micron sized aggregates which are visible by light microscopy. During the first step of our SdFFF particle analysis procedure, the cells are collected, lysed and treated with Proteinase K to digest proteins. Figure 1C shows a microscopy image of the cell debris and aggregated fluorescent particles at this stage of the isolation process. A sample after the final cleanup prior to FFF analysis was analyzed via fluorescence microscopy, however the particles were well dispersed, and not visible, by light microscopy (data not shown). Figure 1D shows a TEM image of these particles after final cleanup and dried onto a grid. The particles are aggregated and coated by residual organic material. SdFFF separation of the particles form soluble components yields the monodispersed particles can be seen by TEM (data not shown) [19]. These rhodamine-labeled SiO_2 _particles have the same manufacturer, and nominal size and surface functionalization as the unlabeled SiO_2 _particles used for the SdFFF experiments.

**Figure 2:**
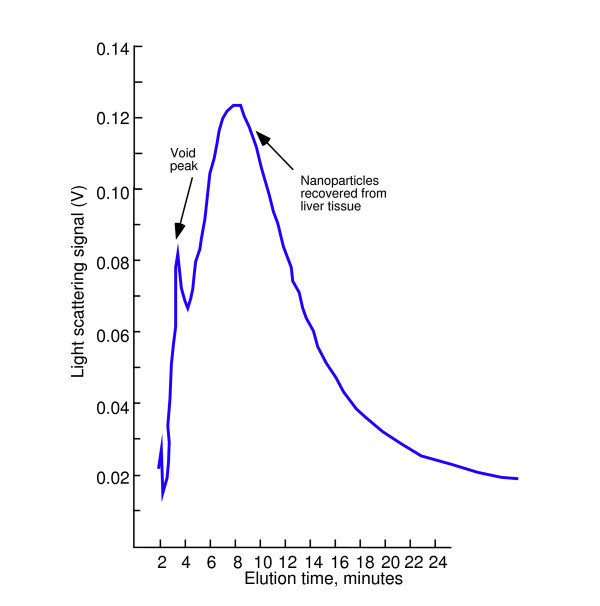
SdFFF fractogram of 70-nm SiO_2 _particles recovered from acid-digested rat liver.

Figure 2 shows a fractogram demonstrating that unlabeled 70-nm particles could be recovered from acid-digested rat liver tissue, sized by SdFFF, and quantified with a light scattering detector. The elution time of the particles in the acid-digested sample agreed with the 70-nm standard in surfactant. A "void peak" is commonly seen at the start of an FFF separation [18]. Caldwell *et al*. describes the contents of this peak as containing soluble components as well as suspended particles small enough to remain uniformly distributed across the channel even in the presence of the field [13]. Liver tissue was used because it is non-fibrous and digestion with concentrated nitric acid resulted in complete digestion of the tissue with the fewest processing steps. Since the nitric acid process is limited to acid-insoluble particles we next developed a more gentle tissue processing protocol utilizing protease enzymes. Lung tissue was used because it is the primary target in particle inhalation studies. Tissue processing method development experiments (data not shown) led to the protocol described in Methods below which involved the use of specific enzymes to digest the extracellular matrix, inclusion of an aqueous surfactant in all processing steps, and sonication to redisperse the particles after centrifugation.

**Figure 3:**
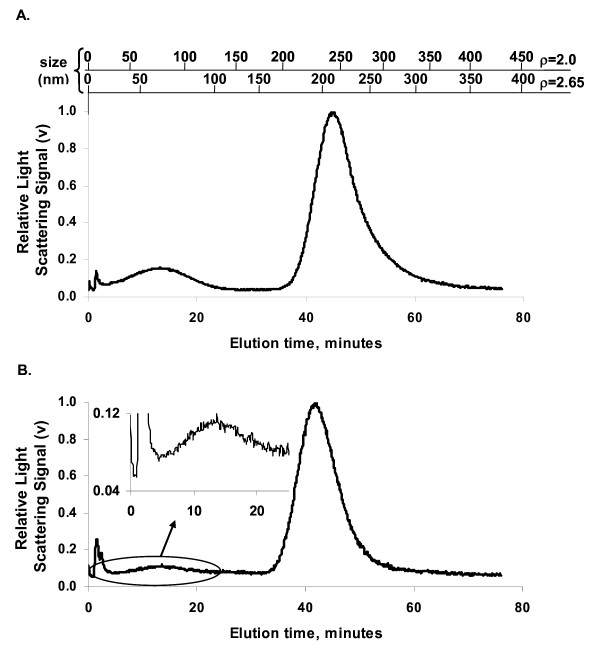
A. SdFFF fractogram of the reference sample of 70 and 250-nm particles mixed in surfactant. The secondary x-axis depicts the theoretical particle size corresponding to the elution time for two particle densities. B. SdFFF fractogram of 70 and 250 nm particles isolated from homogenized lung tissue. The inset graph is the circled area enlarged, emphasizing the 70 nm particle peak.

To demonstrate the capability to distinguish nano-sized (diameter < 100 nm) and submicron particles alone and in lung tissue, mixtures of two different sizes of SiO_2 _particles were added either to aqueous surfactant (reference sample) or added to homogenized lung tissue which was processed by the tissue digestion procedure. Figure 3A shows light scattering versus time from SdFFF analysis of the reference sample normalized to the largest peak. This sample contains the 70- and 250-nm manufactured SiO_2 _particles at a 2:1 mass ratio in aqueous surfactant. Figure 3B shows the enzyme-digested rat lung tissue containing 70- and 250-nm manufactured SiO_2 _particles at the same concentration as in the reference sample (2:1 ratio). The inset graph is a magnified version of the circled area showing that we were clearly able to detect 2 particle sizes from a tissue sample. In both figure 3A and 3B graph shows the expected bimodal distribution of SiO_2 _particles. The difference in the relative sizes of the 70- and 250-nm peaks in the reference samples is due to the size-dependent sensitivity of the light scattering detector. Rayleigh scattering theory predicts that scattering intensity from a single particle varies with the d_p_^6 ^where d_p _is particle geometric diameter. However, the number of particles for a given mass increases inversely with the d_p_^3^, and the mass ratio of the 70 to 250 nm particles was 2:1. Thus the expected ratio of peak areas would be about 23:1. A similar experiment using a mixture of 80-nm and 500-nm particles in enzyme-digested rat lung tissue also produced the expected bimodal fractogram (data not shown). The geometrical size of the 70-nm particles was confirmed by transmission electron microscopy (TEM) of a sample collected after the SdFFF separation [19].

Well established FFF theory [11] allows the particle size corresponding to a given elution time to be calculated directly from first principles [19]. For SdFFF the calculation is based on measurable physical parameters of the apparatus, the carrier fluid, and the particle density, and involves the equations for settling velocity, particle diffusion rate, and laminar flow profile. Figure 3A shows the theoretical particle sizes (top x-axis labels) corresponding to the measured elution time (bottom x-axis labels) for two different particle densities. These assumed densities, 2.65 and 2.0 g/cm^3^, correspond to quartz and the density of the 70-nm particles obtained from the vendor datasheet. These assumed densities span a reasonable range for various amorphous and crystalline forms of SiO_2_. As can be seen from the differences between the two sets of theoretical sizes, the particle size corresponding to a given elution time is not strongly dependent on the assumed density. Thus nanoparticles can be distinguished from micron-sized particles even when the particle composition and density are uncertain. For example, detection of a particle mode within the time range corresponding to SdFFF separation of nano-sized particles for a plausible range of densities would provide useful hypothesis-generating information in a toxicology study of environmental exposures.

Particle recovery for the experiment in figure 3A and 3B can be estimated from the integrated area under the curve for the SdFFF analysis of the tissue sample and the reference sample [19]. Particle recovery in the enzyme digestion processing was 30% for the 250 nm particles and 22% for the 70 nm particles. These recoveries are representative of one set of experimental data.

## Discussion

The goal of this study was to develop a technique that provides detailed information on the size distribution of unlabeled submicron and nano-sized inorganic particles in toxicology samples. Specifically, we wanted to be able show directly by instrumental analysis whether visible particle clusters, such as are shown in Figure 1B, contain nano-sized primary particles. Elemental analysis and radioactive labeled particles provide mass concentration data but not size data. Microscopy-based techniques provide size data only after image analysis of a sufficient sample to get accurate statistics. Manual image analysis is labor intensive and automated image analysis is subject to artifacts from overlapping particles or poor contrast from the background. SdFFF complements the available techniques by providing detailed size distributions from each sample run.

The sample handling methods used this study involved particle dispersion by ultrasound in the presence of a surfactant to demonstrate the presence of a mode corresponding to the primary particle size of the test particle. Characterizing the size distribution of particle aggregates in biological samples is a complex problem that is outside the scope of this pilot study. Particle aggregation is a dynamic process since aggregates are held together by weak surface forces. Aggregate size can be changed by mechanical force as well as by changes in pH, ionic strength, and concentration of surfactants.

A wide range of unlabeled submicron and nano-sized particles of inorganic materials can potentially be detected in tissue samples by the methodology described in this paper. All these proof-of-concept experiments used SiO_2 _particles, but the technique is directly applicable to a much wider range of particle types, size and shape. The requirements for particle detection by our technique are that the particle be sufficiently dense compared to the carrier fluid, have a different index of refraction from the carrier fluid, and be resistant to the reagents used for tissue digestion and sample cleanup. These requirements are met by essentially all inorganic oxides, pure metal, and elemental carbon-based particle types. Non-spherical particles can also be separated using SdFFF, but mathematical prediction of retention time is more complicated than for spheres. A study using rod-shaped aggregates of latex particles showed that the SdFFF separation time is determined by the maximum dimension of the particle rather than by any average size [21]. Thus, this robust methodology is suitable for use in a wide range of particle toxicology studies that involve correlating biological effects with the concentration of nanoparticles in target organs. The particle size distribution information furnished by SdFFF separation will be uniquely applicable to comparisons of the biological effects of solid particles versus the effects of soluble species, a question that cannot be answered by elemental analysis of ashed or acid-digested samples.

The important question of quantifying human lung burden of combustion-generated nanoparticles provides an example of how the sample preparation and SdFFF separation techniques presented in this paper can be used to complement other methods. Vehicle tailpipe emissions contain submicron particles of carbonaceous material from incomplete combustion and metal oxides from fuel and lubricant additives and engine wear. The carbonaceous primary particulate includes both low-volatility compounds, referred to as organic carbon, and essentially non-volatile large polycyclic molecules, referred to as elemental carbon or black carbon. Grigg *et al*. conducted a study that used light microscopy to measure black material in lung macrophages of healthy children and correlated this lung burden with lung function and modeled levels of particulate matter [22]. Light microscopy measures the two-dimensional projected area of particle aggregates. This approach is labor intensive, introduces artifacts from the image analysis, and provides no information on the primary particle size distribution or the composition of the opaque material. Saxena et al. recently published a technique for quantitative estimation of diesel and carbon black particles in lung cells based on adapting the thermal-optical-transmittance analytical technique developed for measuring organic and elemental carbon in air pollution samples [23]. This technique provides quantification of the low-volatility and non-volatile carbon by a precise instrumental analysis method, but again provides no information on the primary particle size distribution or on the metal oxide components. In contrast, SdFFF analysis of tissue provides quantitative information on the particle size distribution after dispersal of the recovered particles by sonication in surfactant. Our tissue processing method has the potential of offering high sensitivity since the centrifugation steps allow concentrating the particles from large volume of digested tissue into a small aliquot for analysis. Sequential collection of samples during a SdFFF run is a well established technique [18]. The collected samples, which represent concentrated and size-segregated fractions of the initial particles, can be further analyzed, for example by transmission electron microscopy or elemental analysis. Compared to the other approaches cited above, the tissue digestion and SdFFF approach presented here provides the ability to analyze particle size distribution in large samples, such as a whole lung, and provides information on both carbonaceous and metal oxide particles. Carbonaceous combustion particles have lower density than the silicon dioxide used in this study, but analysis of carbon black by SdFFF has been demonstrated [24,25]. With additional method development this technique can become a useful tool for studying environmental particle burdens in lungs. Little is known about the background level of naturally formed nanoparticles, and this technique can also be applied to ecosystem studies of nanoparticles in sentinel and food chain organisms.

Considerable future research will be needed to fully realize the potential of our technique for nanoparticle characterization in toxicology studies. Specifically, improvements are needed to reduce particle losses during the enzyme digestion and particle recovery steps of the tissue processing, to make the method sufficiently reproducible, and to permit precise quantification of the nanoparticle burden per weight of original tissue. This paper describes experiments done with relatively high concentrations of the particles because our goal was to demonstrate proof-of-concept. The limit of detection for 70 nm SiO_2 _particles was 25 μg of particles per SdFFF analysis sample using a light scattering detector. This particle concentration in tissue is within the range reported by *in vitro *and *in vivo *nanoparticle toxicity studies. For example, a study of fine and nanoscale quartz particles reported statistically significant responses with an intratracheal instillation dose of 1 mg/kg which equated to an initial burden of about 140 μg of particles per lung [26]. In an inhalation study exposing mice, rats, and hamsters to ultrafine TiO_2_, the particle burden in the lung was 1.6 mg TiO_2_/g of tissue immediately post exposure [27]. Thus, the currently demonstrated enzyme digestion and FFF detection methodology is applicable to nanoparticle toxicology studies that use superphysiological doses. However, improvement in the method sensitivity will be needed for use in toxicology studies that use environmentally relevant particle exposures. The tissue processing protocol involves particle recovery by centrifugation and the centrifugation process intrinsically allows a sample to be concentrated. Processing larger initial tissue samples and recovering the particles in a small final volume is a straightforward way to achieve detection of low particle concentrations in tissue.

## Conclusion

The capability to detect nanoparticles and to distinguish particle size distribution for unlabeled SiO_2 _in a sample of mammalian lung tissue has been demonstrated. We have shown that not only can we detect unlabeled SiO_2 _nanoparticles isolated from rat lung and liver tissue, but more importantly, we distinguished between nano- and submicron-sized particles isolated from the same tissue. The combination of enzyme digestion of tissue with particle sizing by SdFFF is a novel approach that will greatly facilitate measurements of natural and anthropogenic nanoparticles in laboratory toxicology studies, ecological systems, and human populations. This work introduces a new method to characterize the size distribution of unlabeled inorganic particles in tissue which will be useful for studies focused on the neurological and cardiovascular effects of environmental and occupational exposures to an important class of engineered nanomaterials.

## Methods

### Tissue preparation

**Figure 4:**
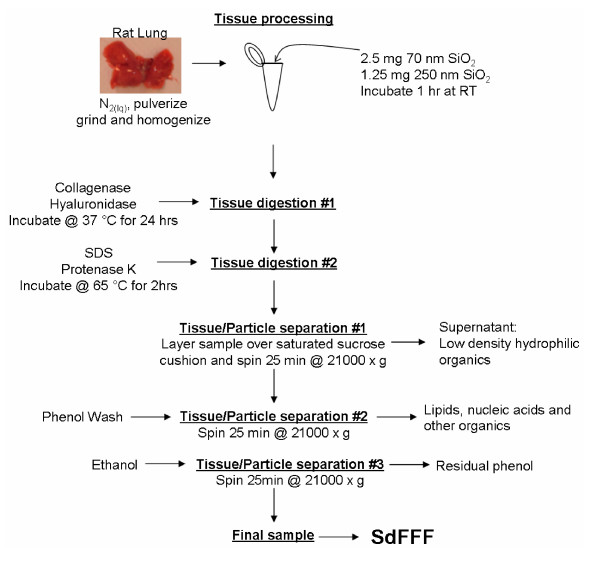
Schematic of the tissue sample preparation protocol.

Figure 4 outlines the tissue preparation, particle addition and isolation, and sample cleanup. Briefly, whole lungs and livers were collected post-mortem from male Sprague-Dawley rats in accordance with an IACUC-approved protocol and snap frozen in liquid nitrogen. The tissue was then ground with a mortar and pestle in liquid nitrogen. The powdered tissue was suspended in 3 ml per gram of tissue of a low-salt buffer (20 mM HEPES, pH 7.9, 25% glycerol, 1.5 mM MgCl_2_, 0.02 M KCl, 0.2 mM EDTA, 0.2 mM phenylmethylsulfonyl fluoride, and 0.5 mM dithiolthreitol). The tissue was further processed by homogenization using a PRO200 series portable homogenizer (ISC BioExpress, Kaysville, UT) at 30,000 rpm until there were no visible chunks and then transferred to a motor-driven Teflon-glass homogenizer (Potter-Elvehjem), (Fisher Scientific) and run at 900 rpm for 2 full passes to ensure the tissue was thoroughly homogenized.

### Nanoparticle addition

We added aliquots of particle suspensions (typically 1–2.5 mg) to homogenized lung or liver tissue. The addition of particles to homogenized tissue demonstrated nanoparticle detection in a complex mixture of biological material without the complications related to *in vivo *particle distribution and uptake and elimination. Particles were 70 nm diameter SiO_2 _(Z-PS-SIL-004-0,07, Postnova Analytics Landsberg, Germany) and 250 nm SiO_2 _(Alfa Aesar, Ward Hill, MA).

### Enzyme digestion

Collagenase (150 U/ml) and hyaluronidase (100 U/ml) (Sigma Aldrich) were added to the homogenized tissue to break up the extracellular matrix. The mixture was incubated overnight at 37°C with shaking. The particle-spiked tissue was then sonicated using an Ultrasonic Processor (Cole-Parmer, Vernon Hills, Illinois) for 20 seconds (2 sec bursts). To further break down the proteins we incubated the tissue with 200 μg/ml Proteinase K (Sigma Aldrich) in 0.5% SDS for 2 hrs at 65°C.

### Particle isolation

The tissue samples were then layered over a saturated sucrose cushion and centrifuged at 21,000 × g for 20 min in a micorcentrifuge (Eppendorf North America, Westbury, NY). The pellets were resuspended in 0.1% FL-70 (Fischer Scientific) and sonicated for 20 sec (2 sec bursts). Phenol was added in a 1:1 (v/v) ratio and incubated with shaking for 5 min followed by another round of centrifugation. The pellet was then resuspended and washed with 70% ethanol and centrifuged. The final pellet was resuspended and sonicated in 0.1% FL-70 with 0.01% sodium azide (Sigma Aldrich).

### SdFFF

Analysis of the final samples was done using a Postnova S101 particle fractionator (Postnova Analytics, Salt Lake City, UT). The injected sample volume was 100 μl using 0.1% FL-70 as the carrier fluid at a rate of 2 ml/min. The initial speed of centrifugation was 1800 rpm and the final speed was 200 rpm. SdFFF run time was typically 90 min. Detection was achieved with a light scattering detector (Brookhaven, Holtsville, NY) at a 90° angle (690 nm laser). A companion paper [19] provides further details of the SdFFF methodology.

### Acid digestion

Acid digestion was used as an alternative to the enzyme digestion protocol above. The tissue sample was transferred to a glass test tube and an equal volume of 60% nitric acid (Fisher Scientific) was added. The test tube was placed in a beaker of hot (94°C) water for about 1 hour or until the tissue was completely digested. The samples were then centrifuged and the pellet washed with dilute acid and finally resuspended in 0.1% of the aqueous surfactant FL-70.

### Cell culture

Treatment of live cell cultures with particles was used as an alternative to adding particles to homogenized tissue, described above. Human aortic endothelial cells (HAEC, Cambrex, Bio Science Walkersville) were cultured in 5% CO_2 _at 37°C in either a T-25 culture flask (Corning, Corning, NY) or a glass bottom culture dish (MatTek Cultureware, Ashland, MA) in endothelial cell growth medium-2 (EGM-2, Cambrex, Bio Science Walkersville) until 90% confluent. Cells were treated by replacing the media with 4 ml of fresh EGM-2 containing 25 μg/cm^2 ^rhodamine-labeled 70 nm SiO_2 _(Z-PS-SIL-RFP-0,07 Postnova Analytics Landsberg, Germany) and incubated for 24 hrs. To harvest the cells and the attached or internalized particles for experiments, the culture medium was removed and the cells were washed with phosphate buffer saline (PBS). Following the removal of PBS, 1 ml TrypLE enzyme (Invitrogen) was added and then removed after one minute and incubated for 5 min. The cells were washed from the dish with fresh media and collected by centrifugation at 200 g, resuspended in 500 μl 0.1% FL-70 and sonicated with a probe for 20 seconds (2 sec bursts). The particles and lysed cell contents were then visualized via fluorescence microscopy and then processed via enzyme digestion starting with the Proteinase K step (Figure 4).

### Fluorescence microscopy

Cells were treated with rhodamine-labeled 70 nm SiO_2 _particles (Z-PS-SIL-RFP-0,07; Postnova Analytics Landsberg, Germany) and fixed in ice cold 100% methanol. The nuclei were stained with DAPI (Molecular Probes). The stained cells were visualized using an Olympus 1 × 50 fluorescent microscope and a Hamamatsu camera. Images were analyzed using ImageJ software.

### Transmission electron microscopy (TEM)

Particle samples for TEM were prepared by washing the particles, concentrating by centrifugation, and resuspending in high-purity water. A 5 μL aliquot was placed on a formvar-coated copper grid and allowed to dry overnight. Samples were imaged on a Philips Techni G2 electron microscope at 100 kV.

## Competing interests

Postnova is a manufacturer of SdFFF instrumentation. CED, JDM, GSY, JMV have no competing financial interests.

## Authors' contributions

CD, JV, GY developed the cell culture and tissue sample preparation methods. SA, ST, and JM provided the Sd-FFF separation methods. All authors participated in the data analysis and manuscript preparation.

## References

[B1] Balbus J, Maynard A, Colvin V, Castranova V, Daston G, Denison R, Dreher K, Goering P, Goldberg A, Kulinowski K, Monteiro-Riviere N, Oberdörster G, Omenn G, Pinkerton K, Ramos K, Rest K, Sass J, Silbergeld E, Wong B (2007). Meeting report: hazard assessment for nanoparticles–report from an interdisciplinary workshop. Environ Health Perspect.

[B2] Gwinn M, Vallyathan V (2006). Nanoparticles: health effects-pros and cons. Environ Health Perspect.

[B3] Schulz H, Harder V, Ibald-Mulli A, Khandoga A, Koenig W, Krombach F, Radykewicz R, Stampfl A, Thorand B, Peters A (2005). Cardiovascular Effects of Fine and Ultrafine Particles. J Aerosol Med.

[B4] Kreyling WG, Semmler M, Erbe F, Mayer P, Takenaka S, Schultz H, Oberdörster G, Ziesenis A (2002). Translocation of ultrafine insoluble iridium particles from lung epithelium to extrapulmonary organs is size dependent but very low. Journal of Toxicology and Environmental Health Part A.

[B5] Peters A, Veronesi B, Calderon-Garciduenas L, Gehr P, Chen LC, Geiser M, Reed W, Rothen-Rutishauser B, Schurch S, Schultz H (2006). Translocation and potential neurological effects of fine and ultrafine particles: a critical update. Part Fibre Toxicol.

[B6] Boxall A, Tiede K, Chaudhry Q (2007). Engineered nanoparticles in soils and water: how do they behave and could they pose a risk to human health. Nanomedicine.

[B7] Sadauskas E, Wallin H, Stoltenberg M, Vogel U, Doering P, Larsen A, Danscher G (2007). Kupffer cells are central in the removal of nanoparticles from the organism. Part Fibre Toxicol.

[B8] Ryman-Rasmussen JP, Riviere JE, Monteiro-Riviere NA (2006). Penetration of intact skin by quantum dots with diverse physicochemical properties. Toxicological Sciences.

[B9] Geiser M, Rothen-Rutishauser B, Kapp N, Schürch S, Kreyling W, Schulz H, Semmler M, Im Hof V, Heyder J, Gehr P (2005). Ultrafine Particles Cross Cellular Membranes by Nonphagocytic Mechanisms in Lungs and in Cultured Cells. Environmental Health Perspectives.

[B10] Elder A, Gelein R, Silva V, Feikert T, Opanashuk L, Carter J, Potter R, Maynard A, Ito Y, Finkelstein J, Oberdörster G (2006). Translocation of Inhaled Ultrafine Manganese Oxide Particles to the Central Nervous System. Environmental Health Perspectives.

[B11] Giddings JC (1991). Unified Separation Science.

[B12] Myers MN (1997). Overview of field-flow fractionation. Journal of Microcolumn Separations.

[B13] Caldwell KD, Compton BJ, Giddings JC, Olson RJ (1984). Sedimentation field-flow fractionation: a method for studying particulates in cataractous lens. Investigative Ophthalmology & Visual Science.

[B14] Baalousha M, Lead JR (2007). Characterization of natural aquatic colloids (< 5 nm) by flow-field flow fractionation and atomic force microscopy. Environ Sci Technol.

[B15] Assemi S, Newcombe G, Hepplewhite C, Beckett R (2004). Characterization of natural organic matter fractions separated by ultrafiltration using flow field-flow fractionation. Water Research.

[B16] Lead JR, Wilkinson KJ, Balnois E, Cutak BJ, Larive CK, Assemi S, Beckett R (2000). Diffusion Coefficients and Polydispersities of the Suwannee River Fulvic Acid: Comparison of Fluorescence Correlation Spectroscopy, Pulsed-Field Gradient Nuclear Magnetic Resonance, and Flow Field-Flow Fractionation. Environ Sci Technol.

[B17] Chun J, Fagan J, Hobbie E, Bauer B (2008). Size separation of single-wall carbon nanotubes by flow-field flow fractionation. Analytical Chemistry.

[B18] Schimph ME, Caldwell KD, Giddings JC, Eds (2000). Field-Flow Fractionation Handbook.

[B19] Takjiki S, Assemi S, Deering CE, Veranth JM, Miller JD (2008). Detection, separation, and quantification of unlabeled silica nanoparticles in biological media using sedimentation field-flow fraction. J Nanoparticle Research accepted for publication.

[B20] Giddings JC, Ratanathanawongs SK, Moon MH (1991). Field-Flow Fractionation: A Versatile Technology for Particle Characterization in the Size Range 10^-3 ^to 10^2 ^micrometers. Kona: Powder and Particle.

[B21] Blau P, Zollars RL (1996). Sedimentation Field-Flow Fractionation of Nonspherical Particles. Journal of Colloid and Interface Science.

[B22] Grigg J, Kulkarni N, Pierse N, Rushton L, O'Callaghan C, Rutman A (2008). Black-Pigmented Material in Airway Macrophages from Healthy Children: Association with Lung Function and Modeled PM10. Res Rep Health Eff Inst.

[B23] Saxena RK, Gilmour MI, Hays MD (2008). Isolation and quantitative estimation of diesel exhaust and carbon black particles ingested by lung epithelial cells and alveolar macrophages in vitro. Bio Techniques.

[B24] Kim Ws, Kim SH, Lee DW, Lee S, Lim CS, Ryu JH (2001). Size Analysis of Automobile Soot Particles Using Field-Flow Fractionation. Environ Sci Technol.

[B25] Park YH, Kim WS, Lee DW (2003). Size analysis of industrial carbon blacks by sedimentation and flow field-flow fractionation. Anal Bioanal Chem.

[B26] Warheit DB, Webb TR, Colvin VL, Reed KL, Sayes CM (2007). Pulmonary bioassay studies with nanoscale and fine-quartz particles in rats: Toxicity is not dependent on particle size but on surface characteristics. Toxicological Sciences.

[B27] Bermudez E, Mangum JB, Wong BA, Asgharian B, Hext PM, Warheit DB, Everitt JI (2004). Pulmonary responses of mice, rats, and hamsters to subchronic inhalation of ultrafine titanium dioxide particles. Toxicological Sciences.

